# Covert gastroduodenal intussusception: a case report

**DOI:** 10.1093/jscr/rjac232

**Published:** 2022-05-18

**Authors:** Aymen Al-Roubaie, Indrajith Withanage

**Affiliations:** Geradlton Regional Hospital, Western Australia, 6530, Australia; Geradlton Regional Hospital, Western Australia, 6530, Australia

## Abstract

Gastroduodenal intussusception is extremely rare and usually symptomatic in adults. Authors here report a case of a 99-year-old female with a gastroduodenal intussusception with no obstructive signs and symptoms or an obvious leading point showed on imaging modality. The index case presented with a three-day history of upper abdominal pain radiated to the back and right shoulder. Examination was unremarkable except for mild tenderness in the epigastric and right hypochondrial areas. A diagnosis of a small gastroduodenal intussusception was made by abdominal CT scan. The patient and family refused gastroscopy. Therefore, a conservative approach was followed, and the patient was discharged home on analgesia after two days of admission in the surgical ward.

## INTRODUCTION

Intussusception refers to the invagination (telescoping) of a part of the intestine into itself [1]. It is more common in children than adults [1]. Although in adult patients, it can be presented with vomiting or fever, abdominal pain is the most common presentation [2].

It accounts for 1–5% of adult with intestinal obstruction with median age of 50 years [1].

Gastroduodenal intussusception is defined as prolapse of part of the stomach through the pylorus to the duodenum. It is usually infrequently diagnosed in the upper part of gastrointestinal tract because of the anatomical relations of these organs which make them less mobile and redundant. Authors report a case of 99 years old lady with a very unusual position of gastroduodenal intussusception, which is extremely rare.

## CASE PRESENTATION

A 99-year-old lady presented to emergency department with a three-day history of upper abdominal pain. The pain was radiated to the back and right shoulder and was described as a dull ache and worsened on the day of the presentation. Neither nausea nor vomiting was noted. The patient was eating and drinking with no issue and was off her food in the day of presenting secondary to pain and abdominal discomfort. There was no change in bowel habits together with no other systemic signs or symptoms has been identified. Her medical history included hiatus hernia, hypothyroidism, pleural effusion, osteoarthritis and asthma. Steroid inhaler and thyroxine were her regular medications only. She does not smoke or drink alcohol and lives in a nursing home. However, she needs assistance with normal daily activities. On clinical examination, abdomen was soft with mild tenderness in the epigastrium and right hypochondrium. There were no signs of guarding, rigidity or distension with normal bowel sounds. Laboratory data showed elevated C reactive protein of 15(normal <5 mg/L) and normal white cell account. Her other blood tests such as hemoglobin, liver function test, lipase, renal function test, lactate and troponin were within normal range.

Abdominal CT scan showed a small intussusception involving the pylorus of the stomach and the first part of the duodenum with approximately 18-mm segment of the pylorus telescoping into the first part of the duodenum. This, however, did not cause a significant obstruction to the gastric outlet and the stomach was collapsed. No lead point mass or lesion identified within the limitations of the study.

**Figure 1:**
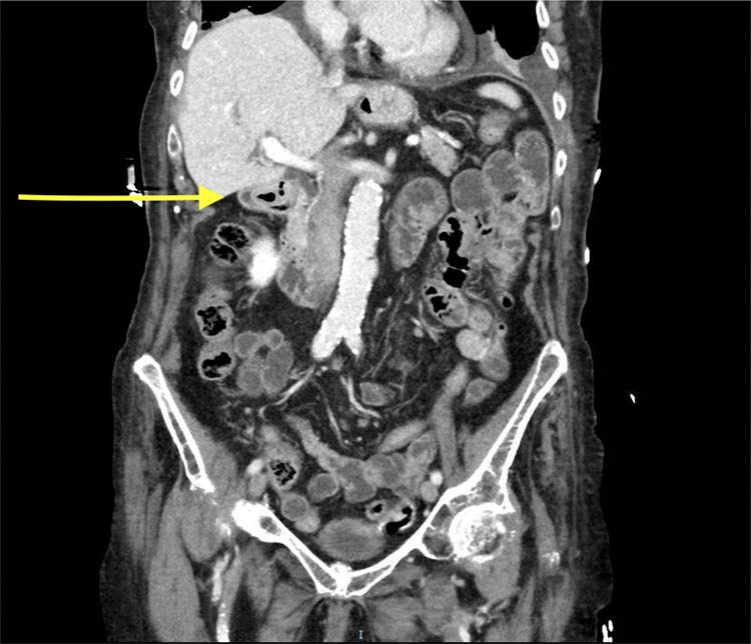
CT scan showing gastroduodenal intussusception (yellow arrow).

**Figure 2:**
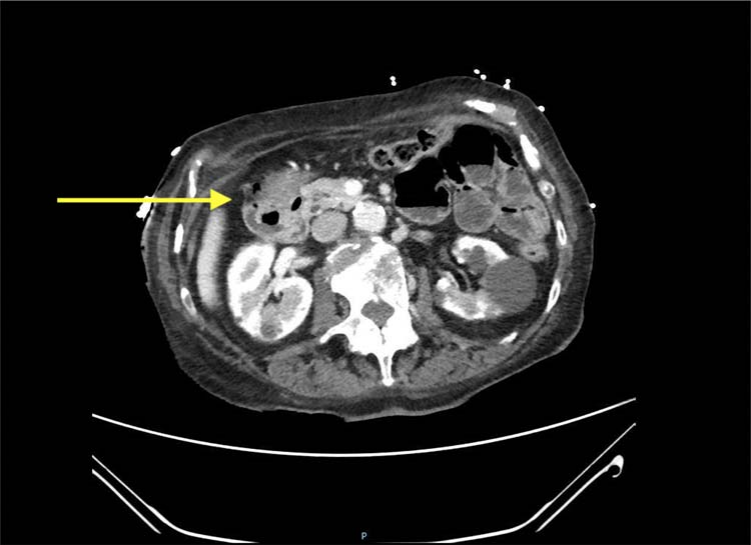
CT scan showing gastroduodenal intussusception (yellow arrow).

The rest of the duodenum and small bowel loops are unremarkable (Figs 1 and 2).

Decision was made to proceed with gastroscopy to confirm diagnosis and try to reduce the intussusception. Nevertheless, the patient refused to have any invasive procedures and expressed her wishes to be treated conservatively only. Therefore, patient was admitted to the surgical ward for pain management and was observed for two days. She was improved with less pain and had no other issues. In a consideration of patient and her family wishes, the patient was discharged home with pain killer and further follow-up at surgical clinic was arranged.

## DISCUSSION

Intussusception happens when there is variation in the intestinal normal peristalsis. It can occur anywhere in the gastrointestinal tract, however, more common in the small bowel, and usually is diagnosed rarely in the large bowel only. Stomach and duodenal intussusception are very rare due to lack of mobility and relatively fixed structures.

The adult intussusception is developed secondary to a leading point in 90% of cases while only 10% of cases have been categorized as idiopathic [3, 4].

Most leading points are benign with a rate of 50–75% especially in the small bowel intussusception [3–6].

Intussusception in adult is usually presented with non-specific abdominal pain rather than the common triad of mass, pain and red jelly stool [7–9].

The pain is the most common adult presentation [8, 10]. Feature of bowel obstruction such as vomiting, and constipation is a common presentation as well [9, 11].

In our case, the patient presented with non-specific abdominal pain with no obstructive symptoms.

The diagnosis depends on history, physical examinations as well as imaging. Although x-rays might be false negative in 20% od cases [12], the ultrasound has almost 100% specificity in diagnosis particularly in children [13]. However, adult intussusception is best diagnosed with Ct scan as the favorable imaging technique [14].

Adult intussusception is traditionally treated by surgery due to the etiology of intussusception is secondary to a leading point; however, recently non-operative approach has gained popularity especially in the setting of radiographic diagnosed intussusception and even in the setting of symptomatic cases [10, 15].

Clinical presentations and imaging findings might help to decide whether to proceed for surgical intervention in patient presented with intestinal obstruction features or there is an identifiable leading mass or in specific kind of intussusception like colocolonic or ileocolic, which associated with high chance of malignancy as a predisposing factor,

Patient who lacks above criteria can be managed conservatively with serial imaging [10, 15, 16]. In our case, patient has no obstructive symptoms together with normal vital signs. Therefore, conservative approach was implemented, and patient has been discharged 2 days later after resolving of her symptoms.

Other researcher has reported treating intussusception conservatively in most idiopathic cases, which shares some of our case clinical scenario [10].

All patients who treated conservatively are followed up to ensure no complication like obstruction has developed. Our case has followed in outpatient surgical clinic in two occasions 14 days apart with no more abdominal pain or any other concerning symptoms.

More cases are required especially in elderly to establish whether conservative approach only is a reliable and safe alternative in special intussusception when they meet certain criteria.

## CONCLUSION

Gastroduodenal intussusception is rare presentation in adults. Author reported a case in a 99-year-old female with gastroduodenal intussusception who was treated conservatively only; however, further cases needed to be studied in order to establish whether conservative approach in elderly with gastroduodenal intussusception could be a solid alternative rather than more invasive laparoscopic or open procedures.
